# Amino acid recognition for automatic resonance assignment of intrinsically disordered proteins

**DOI:** 10.1007/s10858-016-0024-2

**Published:** 2016-02-18

**Authors:** Alessandro Piai, Leonardo Gonnelli, Isabella C. Felli, Roberta Pierattelli, Krzysztof Kazimierczuk, Katarzyna Grudziąż, Wiktor Koźmiński, Anna Zawadzka-Kazimierczuk

**Affiliations:** CERM and Department of Chemistry Ugo Schiff, University of Florence, Via Luigi Sacconi 6, Sesto Fiorentino, 50019 Florence, Italy; Centre of New Technologies, University of Warsaw, Banacha 2C, 02-097 Warsaw, Poland; Faculty of Chemistry, Biological and Chemical Research Centre, University of Warsaw, Żwirki i Wigury 101, 02-089 Warsaw, Poland

**Keywords:** Intrinsically disordered proteins, Automatic assignment, Amino acid-selective experiments, ^13^C direct-detection NMR, High-dimensional NMR experiment, Non-uniform sampling, Compressed sensing, Sparse multidimensional Fourier transform

## Abstract

Resonance assignment is a prerequisite for almost any NMR-based study of proteins. It can be very challenging in some cases, however, due to the nature of the protein under investigation. This is the case with intrinsically disordered proteins, for example, whose NMR spectra suffer from low chemical shifts dispersion and generally low resolution. For these systems, sequence specific assignment is highly time-consuming, so the prospect of using automatic strategies for their assignment is very attractive. In this article we present a new version of the automatic assignment program TSAR dedicated to intrinsically disordered proteins. In particular, we demonstrate how the automatic procedure can be improved by incorporating methods for amino acid recognition and information on chemical shifts in selected amino acids. The approach was tested in silico on 16 disordered proteins and experimentally on α-synuclein, with remarkably good results.

**Electronic supplementary material:**

The online version of this article (doi:10.1007/s10858-016-0024-2) contains supplementary material, which is available to authorized users.

## Introduction

Nuclear magnetic resonance (NMR) is the most powerful method available for studying intrinsically disordered proteins (IDPs) at atomic resolution. It allows us to obtain a variety of information, including structural propensities, dynamics, and interactions with other molecules. But IDPs are rather difficult objects to study with NMR. The high mobility of the polypeptide chain results in exceptionally narrow ranges of chemical shifts. This effect is amplified by the high incidence of sequential repeats; stretches of three or four residues of the same type are often present in IDPs’ sequences. Also, the high abundance of disorder-promoting amino acids and underrepresentation of others (Dunker et al. 2008) contributes to low chemical shifts dispersion. The usually high content of prolines breaks the chains of sequential connectivities obtained via amide proton detected experiments. The combination of the above factors makes the complete sequence-specific resonance assignment of IDPs a challenging task.

High-dimensional (≥4D) experiments (Kazimierczuk et al. 2013; Nowakowski et al. 2015) enable the spreading of cross-peaks over a larger spectral space, and thus better resolution. However, such techniques require the use of non-uniform sampling (NUS) to accelerate data acquisition. Several methods for processing NUS data have been proposed that make it possible to develop experiments of high dimensionality (Mobli and Hoch 2008; Coggins et al. 2010; Orekhov and Jaravine 2011; Freeman and Kupče 2012; Hiller and Wider 2012; Kazimierczuk et al. 2012; Holland and Gladden 2014). These methods have been applied successfully to IDPs. Efforts have also been made to develop carbon direct-detected techniques (Bermel et al. 2009; Felli and Pierattelli 2014), which are invaluable where the fraction of prolines is high or where amide protons undergo fast chemical exchange (Gil et al. 2013). Carbon direct-detected techniques also show superior chemical shift dispersion compared to amide protons-detected experiments (Brutscher et al. 2015). Moreover, these two strategies can be combined: Several ^13^C-detected approaches for high-dimensionality have been proposed (Nováček et al. 2011, 2012; Bermel et al. 2012b; Nováček et al. 2013; Bermel et al. 2013; Dziekański et al. 2015).

During the resonance assignment process, the amino acid types of at least some of the residues must be recognized in order to map the chains of sequentially-linked residues onto the polypeptide. Given a single uniformly-labeled protein sample (e.g. without selective isotope labeling), three main methods are used for this purpose: (1) using the statistical values of chemical shifts for various nuclei of different amino acids; (2) using topological information; and (3) using amino acid type selective experiments.

Method (1) is widely used as usually it does not require additional experiments. C^β^ and H^β^ chemical shifts, which are particularly useful for this purpose, are often recorded within a set of assignment experiments. The statistical values are available from the Biological Magnetic Resonance Bank (BMRB) database (Ulrich et al. 2008), where average C^β^ and H^β^ chemical shifts for each amino acid are calculated based on at least a few thousand chemical shifts. For IDPs, additional statistics are available (Tamiola et al. 2010), which consider not only the residue type but also the residue’s closest neighbors (*i* − 1 and *i* + 1) and are therefore more reliable.

In method (2), the detection of some nuclei limits the range of possible amino acids. For example, the presence of C^β^ chemical shift excludes glycine, the presence of H^N^ chemical shift excludes proline, and the presence of two different H^β^s excludes alanine, isoleucine, threonine, and valine.

Method (3)—amino acid selective experiments—was first proposed by Dötsch and his coworkers (Dötsch et al. 1996a, b, c; Dötsch and Wagner 1996). The approach is based on the triple-resonance CBCA(CO)NH pulse sequence (Grzesiek and Bax 1992), modified to acquire a signal for certain topology-selected amino acid types. The resulting 2D ^1^H–^15^N-HSQC-like spectra contain only resonances originating from the desired amino acid residues.

This concept has since been extensively developed, other researchers adding new selection criteria (Feng et al. 1996; Rios et al. 1996; Schubert et al. 1999, 2000, 2001a, b, c, 2005; Barnwal et al. 2008). The result has been many different strategies, such as the multiplicity selective in-phase coherence transfer (MUSIC) approach developed by Schubert and his collaborators. For selection, several types of pulse sequence components can be employed, including multiple quantum filters (for ^13^CH_n_ or ^15^NH_n_), band-selective pulses on ^13^C and/or ^15^N (for specific nuclei excitation), delay tuning (for choosing the desired coherence transfer pathway), and setting an appropriate number of coherence transfer steps (for choosing side-chains of the desired length). Instead of selecting specific correlations, editing can be implemented and combined with the idea of Hadamard encoding to speed up data collection (Lescop et al. 2008; Pantoja-Uceda and Santoro 2008; Lescop and Brutscher 2009; Feuerstein et al. 2012; Pantoja-Uceda and Santoro 2012). More recently, amino acid selection has also been incorporated into ^13^C-detected experiments (Bertini et al. 2006; Pantoja-Uceda and Santoro 2011; Chakraborty et al. 2012; Jaipuria et al. 2012; Bermel et al. 2012a).

In the current study we show how different amino acid recognition methods can be exploited in automatic resonance assignment, and how the completeness and reliability of the assignment can benefit from this type of information. We present an improved version of the TSAR (Tool for SMFT-based Assignment of Resonances) program (Zawadzka-Kazimierczuk et al. 2012) designed for automatic resonance assignment using experiments of high dimensionality (≥4D). Our improved version includes the information provided by ^13^C-detected amino acid-selective experiments (Bermel et al. 2012a). Additionally, the IDPs’ chemical shifts’ statistics (Tamiola et al. 2010) are incorporated to enable more efficient chain mapping. Finally, we present a small modification of the 4D HCBCACON pulse sequence (Bermel et al. 2012b) in which peaks of residues possessing a single aliphatic C^γ^ carbon are of the opposite sign with respect to that of all other residues.

The approach has been tested in simulations on 16 disordered proteins and experimentally on α-synuclein protein, using both ^1^H-detected (Piai et al. 2014) and ^13^C-detected experiments (Bermel et al. 2012b, 2013) as a source of sequential correlations. To speed up data collection, all spectra were acquired using NUS, making use of recently developed sampling and processing strategies (Kazimierczuk et al. 2009; Kazimierczuk and Orekhov 2011). Data from the high-dimensional experiments was processed using the sparse multidimensional Fourier transform (SMFT) algorithm (Kazimierczuk et al. 2009), whereas data from the 2D amino acid-selective experiments was processed using the compressed sensing (CS) algorithm (Kazimierczuk and Orekhov 2011).

## Materials and methods

All the NMR experiments were performed at 16.4 T on a Bruker Avance spectrometer operating at 700.06 MHz ^1^H, 176.03 MHz ^13^C and 70.94 MHz ^15^N frequencies, equipped with a ^13^C cryogenically cooled probehead optimized for ^13^C-direct detection. A sample of 1.0 mM uniformly ^13^C, ^15^N labeled human α-synuclein in 20 mM phosphate buffer at pH 6.5 was prepared as previously described (Huang et al. 2005). EDTA and NaCl were added to reach the final concentrations of 0.5 and 200 mM respectively, and 10 % D_2_O was added for the lock. All experiments were performed at 285.5 K.

The specific parameters for each amino acid selective experiment are reported in the original publication (Bermel et al. 2012a). Those relating to the γ-selective-HCBCACON experiment are given in the legend of the figure describing the pulse sequence (see Figure S1, Supplementary Material). For ^13^C band-selective *π*/2 and *π* flip angle pulses, Q5 (or time reversed Q5) and Q3 shapes (Emsley and Bodenhausen 1992) with durations of 300 and 220 μs respectively were used, except for *π* pulses that should be band-selective on the C^α^ region (Q3, 860 μs) and for the adiabatic *π* pulse to invert both C′ and C^α^ (smoothed Chirp 500 μs, 25 % smoothing, 80 kHz sweep, 11.3 kHz RF field strength (Bohlen and Bodenhausen 1993)). The ^13^C band selective pulses on C^ali^, C^α^, and C′ were given at the center of each region, and the adiabatic pulse was adjusted to cover the entire ^13^C region.

Decoupling of ^1^H was achieved with waltz16 (Shaka et al. 1983) (1.7 kHz) sequences, and decoupling of ^15^N with garp4 (Shaka et al. 1985) (1.0 kHz) sequences. Each experiment was performed in a pseudo 2D mode, with States method applied in all indirect dimensions to achieve quadrature detection. All experiments employ the IPAP approach to remove splitting in the direct acquisition dimension caused by the homonuclear C^α^–C′ couplings (Bermel et al. 2008).

The experimental parameters are given in Table 1. All experiments were performed using on-grid non-uniform sampling. The “Poisson disk” sampling scheme (Kazimierczuk et al. 2008) was chosen to generate the time schedules with *RSPack* software. All spectra were acquired using *Bruker TopSpin 1.3* software. The experimental data was converted with *nmrPipe* (Delaglio et al. 1995) and then processed using either the *Compressed Sensing* (Kazimierczuk and Orekhov 2011) IRLS algorithm with an iteratively changed lp norm (*p* 1 −> 0) with 30 iterations (2D datasets) or the *Sparse Multidimensional Fourier Transform* (SMFT) (Kazimierczuk et al. 2009) (4D and 5D datasets) implemented in the *Reduced* program. Finally, the *Sparky* program (Goddard and Kneller 2002) was used to display the spectra, and *TSAR* (Zawadzka-Kazimierczuk et al. 2012) was used to assign the resonances. The *RSPack*, *Reduced* and *TSAR* programs are available free of charge for academic users at http://nmr.cent3.uw.edu.pl/software.

**Table 1:** Experimental parameters used in the NMR experiments

	Spectral widths & maximal evolution times				No. of scans	Interscan delay (s)	No. of hyper-complex points	Duration of experiment	Relative data points density (%)
2D A-sel (CA)CON	8800 Hz (^13^C′)	2550 Hz (^15^N) 100.0 ms			12	1.5	40	55 min	15.6
2D A-sel (CA)NCO	8800 Hz (^13^C′)	2550 Hz (^15^N) 31.8 ms			12	1.5	40	55 min	48.8
2D D-sel (CA)CON	8800 Hz (^13^C′)	2550 Hz (^15^N) 100.0 ms			8	1.5	40	40 min	15.6
2D D-sel (CA)NCO	8800 Hz (^13^C′)	2550 Hz (^15^N) 31.8 ms			8	1.5	40	40 min	48.8
2D E-sel (CA)CON	8800 Hz (^13^C′)	2550 Hz (^15^N) 100.0 ms			8	1.5	40	40 min	15.6
2D E-sel (CA)NCO	8800 Hz (^13^C′)	2550 Hz (^15^N) 31.8 ms			8	1.5	40	40 min	48.8
2D FHYW-sel (CA)CON	8800 Hz (^13^C′)	2550 Hz (^15^N) 100.0 ms			16	1.5	40	1 h, 15 min	15.6
2D FHYW-sel (CA)NCO	8800 Hz (^13^C′)	2550 Hz (^15^N) 31.8 ms			16	1.5	40	1 h, 15 min	48.8
2D G-sel (CA)CON	8800 Hz (^13^C′)	2550 Hz (^15^N) 100.0 ms			8	1.5	40	35 min	15.6
2D G-sel (CA)NCO	8800 Hz (^13^C′)	2550 Hz (^15^N) 31.8 ms			8	1.5	40	40 min	48.8
2D N-sel (CA)CON	8800 Hz (^13^C′)	2550 Hz (^15^N) 100.0 ms			8	1.5	40	40 min	15.6
2D N-sel (CA)NCO	8800 Hz (^13^C′)	2550 Hz (^15^N) 31.8 ms			8	1.5	40	40 min	48.8
2D Q-sel (CA)CON	8800 Hz (^13^C′)	2550 Hz (^15^N) 100.0 ms			8	1.5	40	40 min	15.6
2D Q-sel (CA)NCO	8800 Hz (^13^C′)	2550 Hz (^15^N) 31.8 ms			8	1.5	40	40 min	48.8
2D S-sel (CA)CON	8800 Hz (^13^C′)	2550 Hz (^15^N) 49.8 ms			8	1.5	32	30 min	25.0
2D S-sel (CA)NCO	8800 Hz (^13^C′)	2550 Hz (^15^N) 31.8 ms			8	1.5	40	40 min	48.8
4D γ-selective- HCBCACON	8800 Hz (^13^C′)	2550 Hz (^15^N) 60.4 ms	12,500 Hz (^13^C^α,β^) 20.5 ms	5000 Hz (^1^H) 15.0 ms	4	1.1	1540	28 h	0.051

In all experiments the number of complex points in acquisition dimension was set to 512

Experimental parameters for the 3D (H)CACON, 4D HCBCACON, 5D (HCA)CONCACON, 5D HNCACON, 5D (H)CACON(CA)CON, 3D BT-HNCO, 5D BT-(H)NCO(CAN)CONNH, and 5D BT-HN(COCAN)CONNH experiments are presented in the original publications (Bermel et al. 2012b, 2013; Piai et al. 2014)

## Results and discussion

### Methods

The TSAR program (Zawadzka-Kazimierczuk et al. 2012) was developed to analyze data from experiments of high dimensionality processed using the sparse multidimensional Fourier transform (SMFT) algorithm (Kazimierczuk et al. 2009). In this method, instead of computing the full multidimensional spectrum, a set of 2D cross-sections only are calculated. This can be done using the peak list of a lower-dimensional *basisspectrum* that shares some of the dimensions with the high-dimensional spectrum. For each *basis peak*, a single cross-section can usually be calculated. Depending on the type of experiment, each cross-section displays one or more peaks; if the experiment provides sequential connectivities, some peaks are redundant in the cross-sections originating from adjacent residues. Importantly, if several multidimensional spectra have to be analyzed together, they must all be processed using the same basis peak list. The strategy for resonance assignment using this kind of input relies on a comparison of the positions of peaks, creating chains of cross-sections. Recognition of the amino acid type of some of the residues makes it possible to map the cross-sections chains onto the protein sequence, which completes the assignment. In the past, TSAR employed just two of the three methods for amino acid identification described in the Introduction to this paper, namely (1) BMRB chemical shift statistics for C^β^, H^β^, C^α^, H^α^ nuclei, and (2) topological information.

**Fig. 1:**
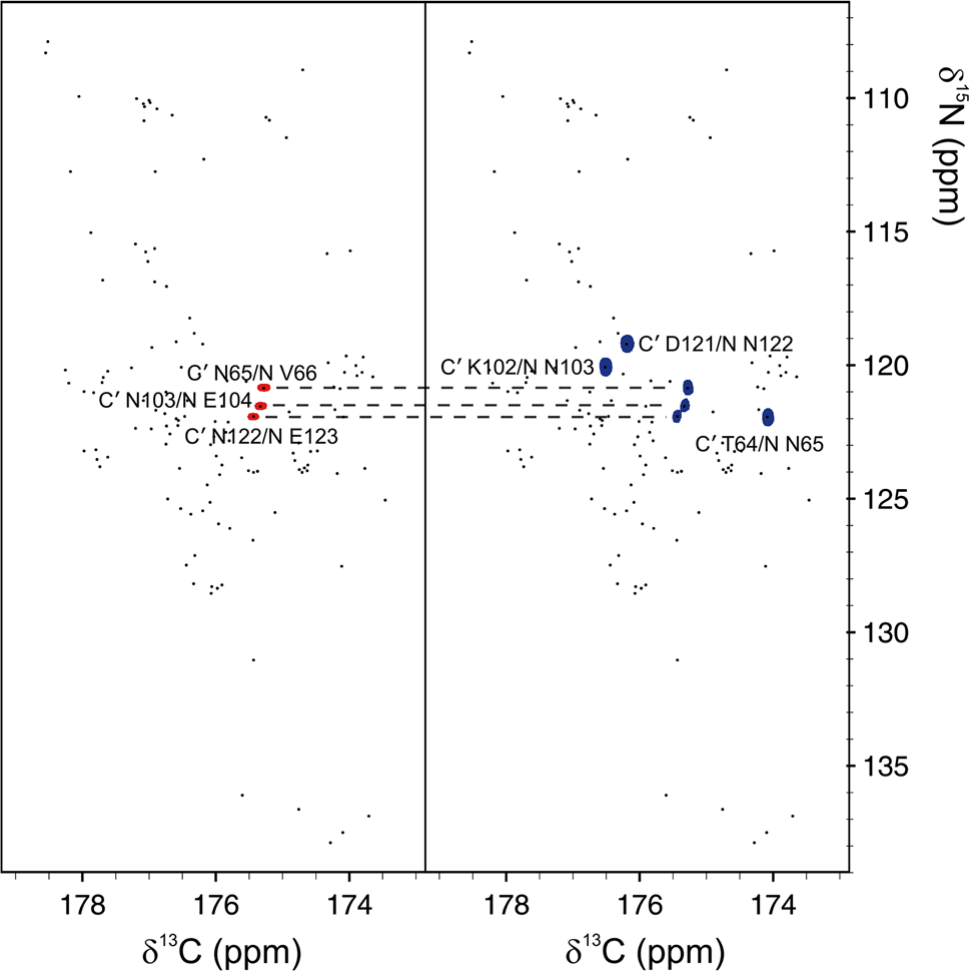
Analysis of amino-acid selective experiments. As an example, asparagine-selective 2D (CA)CON (*left*) and (CA)NCO (*right*) spectra are reported. The basis peak list (*black dots*) is plotted on top of both spectra. For each C′_*i*-*1*_–N_*i*_ cross-peak, the comparison of the two spectra allows us to determine if asparagine residue is at position *i*−*1* or *i*

The main goal of the present work was to implement method (3), i.e. amino acid-selective experiments. Previous TSAR version only exploited the change of the sign of peak intensities in the absence of C^β^ nucleus for glycine residues, which occurs in experiments where C^α^ transverse magnetization evolves for c.a. 1/*J*_Cα–Cβ_. To see if it was possible to achieve automated assignment of highly overlapping IDP resonances, we decided to use 2D spectra with N and C′ dimensions, which provide superior resolution and make it possible to detect prolines. Two types of such spectra are available (Bermel et al. 2012a): CACON-based and CANCO-based. In the 2D (CA)CON amino acid-selective spectrum, a C′_i−1_–N_i_ peak appears if residue *i* − 1 is of the specified type. In the 2D (CA)NCO amino acid-selective spectrum, a C′_i−1_−N_i_ peak appears if residue *i* − 1 or residue *i* is of that type. Comparing the two spectra allows us to discriminate C′_i−1_–N_i_ peaks related to *i* − 1 or *i* residues (Fig. 1).

**Fig. 2:**
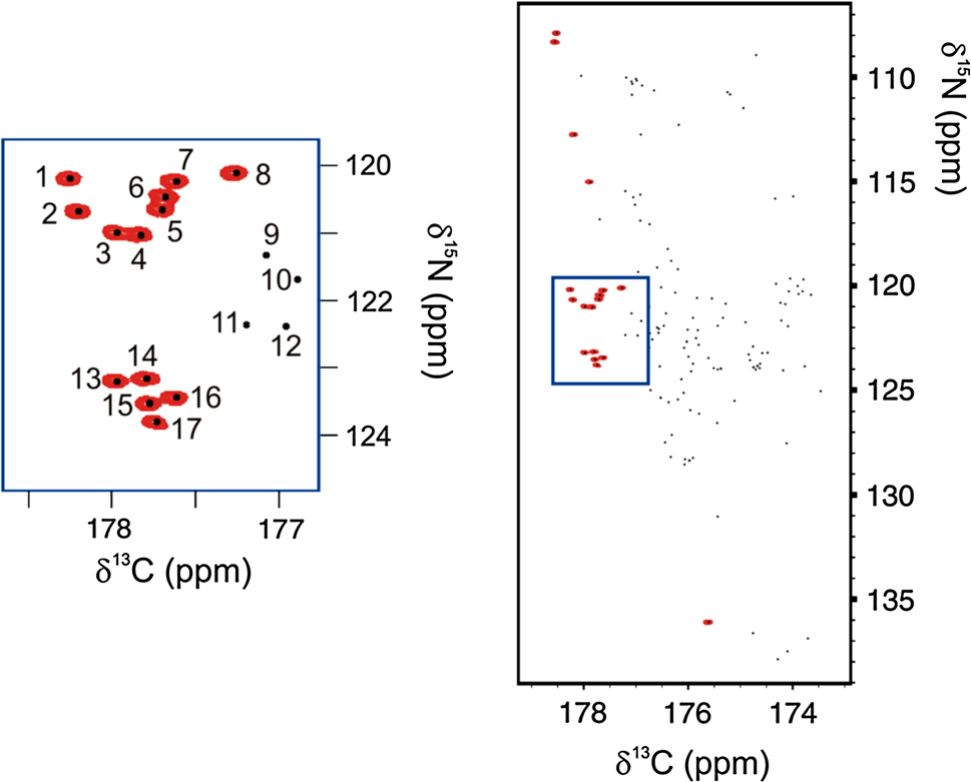
Alanine-selective 2D (CA)CON spectrum (*red*) with the basis peak list superimposed (*black dots*). On the *left*, the close-up view of the spectral region inside the *blue square* is reported. In the example, during the automatic assignment stage, basis peaks on top of the NMR signals are known by TSAR to be related to alanine residues. To make the picture clearer, the number of each basis peak is shown only in the spectral region extracted on the *left*

To use such experiments in parallel with SMFT-processed high-dimensional data, the basis peaks corresponding to the selected amino acids must be found. This can be done if the dimensions of the amino acid-selective experiments—in our case amide nitrogen and carbonyl carbon dimensions—are also present in the basis spectrum. The basis peak list can then be plotted on the amino acid-selective spectrum and the basis peaks corresponding to the given amino acid easily identified (Fig. 2). Information on these basis peaks numbers can then be fed into the TSAR program to support the assignment process (for the format of TSAR input files, see Supplementary Material). Of course, it may happen that two basis peaks show up at the position of the amino acid-selective spectrum peak, for example if they are overlapping, or if the amino acid-selective spectrum is not resolved enough. This makes the task more difficult, but TSAR is still able to manage its task. As mentioned above, the only requirement for combining SMFT-processed data with the amino acid-selective data is to have C′ and N dimensions in the basis spectrum. Therefore, although (CA)CON and (CA)NCO experiments exploit carbon detection, they can be combined with both carbon-detected (e.g. with 3D CACON basis spectrum) and also with proton-detected (e.g. with 3D HNCO basis spectrum) experiments for resonance assignment, which makes them even more generally applicable.

In this study we also make use of another method for obtaining amino acid-related information. We modify the 4D HCBCACON pulse sequence (Bermel et al. 2012b) so that the signs of the cross-peaks depend on the topological properties of the residue. The delay for C^β^ scalar coupling evolution is increased to 21.0 ms (see Supplementary Material), which allows us to keep the efficiency of the coherence transfer (additional relaxation losses can be neglected for IDPs) and at the same time reverse peak signs for some residues. If a residue *i* possesses exactly one aliphatic C^γ^ carbon (E, K, L, M, P, Q, R and T residues), then the H_i_^β^–C_i_^β^ peak has the opposite sign to that of all the other residues. Such zero–one information adds to C^β^ and H^β^ chemical shifts values, which was the only information provided by the 4D HCBCACON experiment published earlier (Bermel et al. 2012b). It thus improves the performance of the automated assignment. The new pulse sequence also allows us to unambiguously distinguish some residues possessing C^β^ and H^β^ chemical shifts which may be not so different: S can easily be discriminated from T, I can be easily discriminated from L, and V can easily be discriminated from E, K, M, P, Q, and R. Additionally, the extension of the C^α/β^ chemical shift evolution increases the resolution of that dimension, with consequent benefits for the comparison of cross-peak spectral positions performed by TSAR. In the old version, TSAR was prepared for single amino acid recognition by peak sign, which was used only for glycine. The current software version accepts sign change in the presence of a user-defined set of residues.

The final major change enabling more efficient amino acid recognition in TSAR is to incorporate the statistical C^α^ and C^β^ chemical shift values calculated using a set of IDPs (Tamiola et al. 2010). TSAR uses statistical chemical shift values at two stages of the operation: (a) recognition of possible amino acids for a single plane, before forming cross-sections chains; and (b) cross-sections chains mapping. During stage (a), the working procedure of TSAR implies that if some chemical shifts exceed the statistical average with four standard deviations for certain amino acids, then these amino acids are excluded from the range of possible ones. During stage (b), if one chain matches in a few sites or a few chains of similar length fit into one site, the deviation from the statistical values in units of standard deviations is calculated, and if the best score is at least three times smaller than the second best, then the better chain is assigned. In the new TSAR version, during stage (b) the chain length is also considered. If up to three C^β^ chemical shifts are known, then the better chain is assigned only if the deviation score is at least ten times smaller than the second best.

In the previous software version of TSAR, the BMRB values were used at both stages. In the version of TSAR presented here, the IDP-specific values (Tamiola et al. 2010) are used at stage (b). Such values cannot be employed during stage (a) when the neighboring residues are not yet known, as these values depend on the preceding and following residue type. Thus, at the stage of amino acid recognition for a single plane, the BMRB values are still used. Nonetheless, some IDP-oriented changes were also made at this stage: It was discovered that for IDPs it is better to exclude an amino acid if the chemical shift exceeds two (rather than four) standard deviations. Additionally, C^α^ chemical shifts were incorporated for amino-acid recognition, while previously only glycine residues could be identified based exclusively on C^α^ chemical shifts.

### Simulations

The new methods of amino acid recognition were tested in a set of simulations. Our aim was to verify the agreement of the statistical values used in the new TSAR version with the real chemical shifts of unstructured proteins. Also, by using the simulations we wanted to check whether the 4D γ-selective—HCBCACON provided information that improves the result of the assignment. An additional goal was to evaluate the effectiveness of incorporating the data from amino-acid selective experiments.

Sixteen proteins (see Table 2) were chosen from the BMRB database. Their lengths ranged from 26 to 467 amino-acid residues. Thirteen of them were natively unstructured, while the remaining three (BMRB IDs 15201, 16626 and 16627) were urea-unfolded proteins. Importantly, none of the proteins chosen by us, except for α-synuclein, was used to prepare the IDPs’ statistics (Tamiola et al. 2010). One of the proteins (BMRB ID 16912) is partially folded, so in the simulations we just used its unstructured C-terminal fragment (residues 79Ser-172Lys). For each of the proteins, peak lists relative to six ^13^C-detected experiments were generated using the chemical shifts deposited in BMRB. The experiments included 3D CACON, 5D (H)CACON(CA)CON (Bermel et al. 2013), 5D HNCACON (Bermel et al. 2012b), 5D (HCA)CONCACON (Bermel et al. 2013), 4D HCBCACON (Bermel et al. 2012b), and 4D γ-selective-HCBCACON. The peak lists for the 4D and 5D experiments were generated in a format accepted by TSAR: The positions of the peaks in the two dimensions not fixed for SMFT only were given, together with information about the corresponding basis peak. For all proteins, the data from amino-acid selective experiments was also generated.

**Table 2:** Proteins used for simulations and number of residues of each of the types selected in amino-acid selective experiments

BMRB ID	Sequence length	No. of residues of each type								Percentage of residues detected by aa-selective experiments used in datasets 6
		E	G	A	Q^a^	F/H/Y/W	S	N	D	
6968	140	**18**	**18**	**19**	**9**	6	4	3	6	39.3
11526	148	**10**	8	4	**20**	**10**	**13**	7	**11**	43.2
15176	120	**14**	6	6	**11**	**10**	**15**	3	3	41.7
15179	159	**19**	8	**13**	11	11	**14**	5	**18**	40.3
15201	148	6	**20**	10	**15**	**26**	13	8	8	41.2
15883	92	**9**	**6**	**12**	**7**	**5**	3	2	2	42.4
16445	48	**5**	**6**	**2**	**6**	1	2	0	1	39.6
16626	76	**6**	**6**	2	**8**	**4**	3	2	**5**	38.2
16627	56	**5**	4	**6**	**5**	**6**	0	3	5	39.3
16912^b^	94	**25**	0	4	2	6	1	0	**27**	55.3
17325	66	2	**4**	4	**6**	**5**	**11**	4	4	39.4
18417	253	**44**	18	**58**	12	10	16	4	10	40.3
18580	130	**13**	1	7	**17**	**15**	8	**10**	6	42.3
18851	26	1	1	**7**	**3**	1	0	1	0	38.5
18895	141	**11**	**10**	**17**	4	3	**17**	3	5	39.0
19135	467	**36**	**39**	**44**	**34**	23	**58**	15	17	45.2

Numbers of the amino acid types used in datasets A6, B6 and C6 appear in bold

^a^As shown in the section Experimental results, in the Q-selective experiment N residues also appear, thus the numbers of these two residues were added here

^b^The protein is partially structured; the unstructured C-terminal fragment only (residues 79Ser-172Lys) was used for simulations

The artificial data was slightly perturbed: The peak positions were jittered and peak overlap was included. The latter perturbation in particular was realized in three different ways. First, in high-dimensional peak lists the peaks of similar coordinates were joined into a single peak. Second, cross-sections corresponding to overlapping basis peaks were also overlapping: Peaks originating from both overlapping basis peaks appeared on both cross-sections. And third, peak overlap was also considered during the generation of data from amino-acid selective experiments, leading to some ambiguity. The level of perturbations in each of the above aspects was similar to that found for the real IDP data (α-synuclein sample), so the quality of data is similar to the quality of data in the case of real proteins.

In real cases there is an additional source of data imperfection: The dataset is typically incomplete, i.e. some peaks are missing. The level of completeness of the data relates to many factors, including protein concentration, measurement time, pulse sequence efficiency, relaxation rates, and exchange rates. For the proteins used in the simulations, the level of completeness of the data deposited in BMRB ranged from 84.9 to 100 % of the protein residues (excluding the first residue, for which there is no basis peak). The residue was considered to be “present” if all the chemical shifts of the corresponding basis peak were known. As a result, for some “present” residues, certain resonances were unknown.

Several datasets were constructed for each protein. Each dataset included the basis spectrum 3D CACON and one, two or three 5D spectra providing the sequential connectivities. Various numbers of linking experiments were used, due to the fact that the TSAR program forms cross-sections chains whose lengths depend on the quality of the experiments providing sequential correlations. Thus, the datasets containing different combinations of such experiments allow us to evaluate the efficiency of amino-acid recognition methods for various chain lengths. In datasets A1–A6, the linking experiment was 5D (H)CACON(CA)CON. In datasets B1–B6, they were 5D (H)CACON(CA)CON and 5D HNCACON. In datasets C1–C6, the connectivities were provided by 5D (H)CACON(CA)CON, 5D HNCACON, and 5D (HCA)CONCACON spectra. For protein 19135, due to the lack of information about H^N^ chemical shifts in the BMRB entry, the experiments providing the sequential connectivities were (H)CACON(CA)CON and (HCA)CONCACON for datasets B1–B6. Datasets C1–C6 were not constructed in this case.

For amino acid recognition, in some datasets either the 4D HCBCACON (Bermel et al. 2012b) or the new 4D γ-selective-HCBCACON experiments yielding C^β^ and H^β^ chemical shifts was employed, while in others the information provided by the amino acid-selective experiments was used. The latter group consisted of ^13^C-detected 2D (CA)CON- and (CA)NCO-based amino acid selective experiments (Bermel et al. 2012a), including the following selections: A, D, E, FHYW, G, N, Q, and S. Datasets A1, B1, and C1 did not contain any additional information on amino acids. Datasets A2, B2 and C2 exploited the 4D HCBCACON experiment. Datasets A3, B3, and C3 used the 4D γ-selective-HCBCACON experiment, which carries extra information in the peak signs. Datasets A4, B4, and C4 employed all eight amino-acid selective 2D experiments (selecting A, D, E, G, N, Q, S, and FHYW), both in the (CA)CON and (CA)NCO versions. Datasets A5, B5, and C5 employed all 2D amino acid selective experiments, but in the (CA)CON version only. Datasets A6, B6, and C6 exploited some of the amino-acid selective experiments in both the (CA)CON and (CA)NCO versions. The choice of amino-acids to be selected was based on the sequence of each protein (see Table 2), such that approximately 40 % of residues were extracted from the total. This meant that the numbers of aa-selective experiments varied from two (as for protein 16912) to five (as for protein 15883), depending on the abundances of different amino acids in a given protein.

The datasets were analyzed using the new version of the TSAR program, and the results compared with the original BMRB assignment. Additionally, datasets A1, B1, C1, A2, B2, and C2 were analyzed using the old version of the TSAR program, to compare the efficiency of the amino-acid recognition procedures in the old and new versions. Datasets 3, 4, 5, and 6 are not accepted by the old version of TSAR. The results are presented in Table 3 (datasets A1–A6), Table 4 (datasets B1–B6), and Table 5 (datasets C1–C6).

**Table 3:** Automatic assignment results for simulated data—one linking experiment

BMRB ID	Percentage of residues present in BMRB (in parenthesis: in long/short TSAR chains)	Percentage of correctly/incorrectly assigned residues					
		Dataset A1	Dataset A2	Dataset A3	Dataset A4	Dataset A5	Dataset A6
6968	100 (69.1/30.9)	84.2/0.0 (32.4/0.0)	87.1/0.0 (86.3/0.0)	87.1/0.0	90.6/0.0	87.8/0.0	88.5/0.0
11526	94.6 (71.4/23.1)	43.5/0.0 (30.6/14.3)	70.7/2.7 (88.4/0.0)	87.8/2.0	90.5/0.7	76.9/2.7	89.8/0.7
15176	84.9 (55.5/29.4)	24.4/0.0 (14.3/1.7)	71.4/0.0 (69.7/2.5)	71.4/0.0	78.2/0.0	71.4/0.0	74.8/0.0
15179	89.2 (72.2/17.1)	29.7/0.0 (0.0/0.0)	79.7/0.0 (78.5/0.6)	79.7/0.0	84.2/0.0	81.6/0.6	79.1/0.0
15201^a^	97.3 (60.5/36.7)	33.3/0.0 (19.0/2.7)	87.8/0.0 (72.1/6.1)	87.8/0.0	88.4/0.0	85.7/0.0	65.3/0.0
15883	95.6 (87.9/7.7)	93.4/0.0 (71.4/22.0)	94.5/0.0 (94.5/0.0)	94.5/0.0	93.4/0.0	93.4/0.0	93.4/0.0
16445	87.2 (40.4/46.8)	0.0/0.0 (0.0/0.0)	12.8/0.0 (38.3/12.8)	12.8/0.0	23.4/0.0	21.3/0.0	21.3/0.0
16626^a^	94.7 (84.0/10.7)	93.3/0.0 (21.3/0.0)	90.7/0.0 (93.3/0.0)	90.7/0.0	94.7/0.0	93.3/0.0	94.7/0.0
16627^a^	100.0 (92.7/7.3)	96.4/0.0 (72.7/0.0)	96.4/0.0 (92.7/0.0)	96.4/0.0	96.4/0.0	96.4/0.0	96.4/0.0
16912^b^	100.0 (31.2/68.8)	7.5/0.0 (0.0/7.5)	31.2/2.2 (25.8/3.2)	31.2/2.2	35.5/0.0	34.4/1.1	25.8/1.1
17325	86.2 (72.3/13.8)	20.0/0.0 (20.0/0.0)	86.2/0.0 (84.6/0.0)	86.2/0.0	76.9/0.0	64.6/0.0	75.4/0.0
18417	91.3 (15.5/75.8)	4.0/0.0 (0.0/0.8)	17.9/1.6 (13.5/13.9)	19.8/1.6	40.5/0.4	22.2/1.6	7.9/0.0
18580	86.0 (53.5/32.6)	23.3/0.0 (0.0/0.0)	54.3/1.6 (45.7/3.9)	51.9/0.0	77.5/0.0	64.3/0.8	67.4/0.8
18851	92.0 (64.0/28.0)	0.0/0.0 (0.0/0.0)	60.0/4.0 (60.0/4.0)	60.0/4.0	72.0/0.0	60.0/4.0	56.0/0.0
18895	89.2 (47.1/41.4)	19.3/0.7 (0.0/15.7)	62.1/0.0 (64.3/0.0)	66.4/0.0	72.1/0.0	57.1/0.0	62.9/0.7
19135	99.8 (31.5/68.2)	4.1/0.0 (0.0/2.4)	47.4/0.9 (33.3/21.2)	48.9/0.9	57.3/4.7	37.8/1.9	36.5/0.6

^a^The protein was urea-unfolded

^b^The protein is partially structured; the unstructured C-terminal fragment only (residues 79Ser-172Lys) was used for simulations

**Table 4:** Automatic assignment results for simulated data—two linking experiments

BMRB ID	Percentage of residues present in BMRB (in parenthesis: in long/short TSAR chains)	Percentage of correctly/incorrectly assigned residues					
		Dataset B1	Dataset B2	Dataset B3	Dataset B4	Dataset B5	Dataset B6
6968	100 (94.2/5.8)	94.2/0.0 (94.2/0.0)	94.2/0.0 (94.2/0.0)	94.2/0.0	94.2/0.0	94.2/0.0	94.2/0.0
11526	94.6 (89.1/5.4)	93.9/0.0 (78.9/12.9)	92.5/0.0 (94.6/0.0)	93.2/0.0	93.9/0.0	93.9/0.0	93.9/0.0
15176	84.9 (68.9/16.0)	63.9/0.0 (19.3/14.3)	79.8/0.0 (72.3/3.4)	79.8/0.0	83.2/0.0	79.8/0.0	79.8/0.0
15179	89.2 (79.7/9.5)	73.4/0.0 (7.0/9.5)	87.3/0.0 (86.7/0.6)	87.3/0.0	86.1/0.0	86.1/0.0	85.4/0.0
15201^a^	97.3 (89.1/8.2)	83.0/0.0 (86.4/1.4)	95.9/0.0 (91.2/2.0)	95.9/0.0	95.9/0.0	95.9/0.0	95.9/0.0
15883	95.6 (95.6/0.0)	95.6/0.0 (95.6/0.0)	95.6/0.0 (95.6/0.0)	95.6/0.0	95.6/0.0	95.6/0.0	95.6/0.0
16445	87.2 (51.1/36.2)	42.6/0.0 (0.0/0.0)	68.1/0.0 (70.2/12.8)	68.1/0.0	83.0/0.0	83.0/0.0	83.0/0.0
16626^a^	94.7 (90.7/4.0)	94.7/0.0 (94.7/0.0)	92.0/0.0 (94.7/0.0)	92.0/0.0	94.7/0.0	94.7/0.0	94.7/0.0
16627^a^	100.0 (92.7/7.3)	98.2/0.0 (98.2/0.0)	98.2/0.0 (98.2/0.0)	98.2/0.0	98.2/0.0	98.2/0.0	98.2/0.0
16912^b^	100.0 (87.1/12.9)	46.2/0.0 (0.0/0.0)	89.2/0.0 (89.2/0.0)	89.2/0.0	89.2/2.2	89.2/0.0	89.2/0.0
17325	86.2 (61.5/24.6)	41.5/0.0 (24.6/0.0)	86.2/0.0 (86.2/0.0)	86.2/0.0	86.2/0.0	86.2/0.0	86.2/0.0
18417	91.3 (61.1/30.2)	50.0/0.4 (40.1/0.0)	67.9/0.8 (69.0/0.8)	67.9/0.8	74.6/1.6	72.2/1.6	68.7/0.4
18580	86.0 (77.5/8.5)	35.7/0.0 (10.1/7.0)	82.2/0.0 (82.2/0.0)	82.2/0.0	82.9/0.0	82.2/0.0	82.9/0.0
18851	92.0 (60.0/32.0)	16.0/0.0 (0.0/24.0)	84.0/0.0 (84.0/0.0)	84.0/0.0	84.0/0.0	84.0/0.0	84.0/0.0
18895	89.2 (72.9/15.7)	76.4/1.4 (52.1/25.0)	84.3/0.0 (84.3/0.0)	84.3/0.0	86.4/0.0	83.6/0.0	82.1/1.4
19135^c^	99.8 (79.6/20.2)	66.1/0.0 (27.3/4.5)	91.8/0.2 (86.3/1.9)	91.8/0.2	90.8/0.6	87.8/1.1	89.9/0.0

^a^The protein was urea-unfolded

^b^The protein is partially structured; the unstructured C-terminal fragment only (residues 79Ser-172Lys) was used for simulations

^c^This BMRB deposition lack H^N^ chemical shifts, so for simulations the 5D HNCACON data was replaced with the 5D (HCA)CONCACON

**Table 5:** Automatic assignment results for simulated data—three linking experiments

BMRB ID	Percentage of residues present in BMRB (in parenthesis: in long/short TSAR chains)	Percentage of correctly/incorrectly assigned residues					
		Dataset C1	Dataset C2	Dataset C3	Dataset C4	Dataset C5	Dataset C6
6968	100 (94.2/5.8)	94.2/0.0 (94.2/0.0)	94.2/0.0 (94.2/0.0)	94.2/0.0	94.2/0.0	94.2/0.0	94.2/0.0
11526	94.6 (89.1/5.4)	90.5/0.0 (65.3/19.7)	91.8/0.0 (93.9/0.0)	92.5/0.0	93.2/0.0	93.2/0.0	93.2/0.0
15176	84.9 (65.5/19.3)	28.6/0.0 (20.2/10.1)	81.5/0.0 (76.5/1.7)	81.5/0.0	84.0/0.0	81.5/0.0	81.5/0.0
15179	89.2 (78.5/10.8)	77.2/0.0 (29.1/34.8)	86.7/0.6 (86.1/1.3)	86.7/0.6	84.8/0.0	85.4/0.0	84.2/0.0
15201^a^	97.3 (87.1/10.2)	81.6/0.0 (83.7/4.1)	95.9/0.0 (90.5/2.0)	95.9/0.0	95.9/0.0	95.9/0.0	95.9/0.0
15883	95.6 (95.6/0.0)	95.6/0.0 (95.6/0.0)	95.6/0.0 (95.6/0.0)	95.6/0.0	95.6/0.0	95.6/0.0	95.6/0.0
16445	87.2 (57.4/29.8)	48.9/0.0 (0.0/0.0)	74.5/0.0 (70.2/12.8)	74.5/0.0	83.0/0.0	83.0/0.0	83.0/0.0
16626^a^	94.7 (90.7/4.0)	94.7/0.0 (94.7/0.0)	92.0/0.0 (94.7/0.0)	92.0/0.0	94.7/0.0	94.7/0.0	94.7/0.0
16627^a^	100.0 (98.2/1.8)	98.2/0.0 (98.2/0.0)	98.2/0.0 (98.2/0.0)	98.2/0.0	98.2/0.0	98.2/0.0	98.2/0.0
16912^b^	100.0 (92.5/7.5)	44.1/0.0 (0.0/0.0)	93.5/0.0 (92.5/0.0)	93.5/0.0	91.4/0.0	93.5/0.0	93.5/0.0
17325	86.2 (69.2/16.9)	56.9/0.0 (44.6/0.0)	86.2/0.0 (86.2/0.0)	86.2/0.0	86.2/0.0	86.2/0.0	86.2/0.0
18417	91.3 (73.0/18.3)	57.5/0.8 (30.6/19.4)	77.8/2.0 (77.8/1.2)	77.8/2.0	80.6/1.2	78.6/1.2	74.2/0.4
18580	86.0 (79.8/6.2)	72.9/0.0 (46.5/7.0)	84.5/0.0 (84.5/0.0)	84.5/0.0	84.5/0.0	84.5/0.0	84.5/0.0
18851	92.0 (60.0/32.0)	16.0/0.0 (0.0/24.0)	84.0/0.0 (84.0/0.0)	84.0/0.0	84.0/0.0	84.0/0.0	84.0/0.0
18895	89.2 (82.1/6.4)	81.4/0.0 (67.1/10.7)	87.9/0.0 (87.1/0.0)	87.9/0.0	88.6/0.0	87.9/0.0	87.9/0.0
19135^c^	99.8	n.a.	n.a.	n.a.	n.a.	n.a.	n.a.

^a^The protein was urea-unfolded

^b^The protein is partially structured; the unstructured C-terminal fragment only (residues 79Ser-172Lys) was used for simulations

^c^This BMRB deposition lacks H^N^ chemical shifts, so the simulation with three connectivities-yielding experiments could not be performed

To assess the data, we need to consider the lengths of the cross-sections chains formed by TSAR. The longer the chain, the easier and more reliable its mapping onto the protein sequence. Chains were divided into two groups: long (≥4 cross-sections) and short (1–3 cross-sections). The length of the chains is influenced by several factors—not just the protein size, but also the chemical shift dispersion, the number of missing basis peaks, the number and quality of connectivity-yielding experiments, and the number of prolines (in the case of H^N^-detected experiments). As can be seen by comparing datasets A, B, and C, the proportion of cross-sections within the long chains generally increases in line with the number of linking experiments (see Tables 3, 4 and 5). This reflects different levels of assignment difficulty for the datasets A, B, and C.

Another factor also influences the complexity of the assignment process: the incidence of repeats in the sequence of proteins. This includes overrepresentation of certain amino acid in the sequence, stretches of several residues of the same amino acids, and multiple occurrences of certain sequential motifs. This factor is more difficult to measure, but should be considered when interpreting the data. In this respect, protein 18851, which contains 11 Arg residues within its 26-residues-long sequence (including one four-Arg stretch), is considered difficult to assign despite its small size.

Several conclusions can be drawn from the resulting data. First, it is evident that providing any type of information on amino acids significantly improves the result of the assignment (datasets 2, 3, 4, 5, and 6 vs. datasets 1). This is not surprising, as the mapping of the cross-sections chains onto the protein sequence is performed using amino acid recognition. For instance, for the protein 15179, the result for dataset A1 is 29.7 % correct assignments, which can be increased to 84.2 % correct assignments for the dataset A4. Where there are only a few cross-sections in short chains (typically below 10 %), they can often be correctly mapped onto the sequence even without the additional information on amino acids. This is true for protein 15883, for example, where 95.6 % correct assignments were obtained for all datasets B1–B6, the maximum possible taking into consideration the completeness of the data. If the chains are very short (typically over 40 % of cross-sections within short chains), extra information on amino acids is beneficial, but the results may still not be satisfactory. This is true for protein 16912 datasets A, for example, where the result can be improved from 7.5 % (A1) to 35.5 % (A4), but is still much too low. In such cases, more experiments providing the sequential connectivities are required (see datasets B and C for protein 16912).

Comparing the results obtained with the old and new versions of TSAR (datasets A1, B1, C1, A2, B2, and C2), we find that the new version generates more reliable results: The proportion of incorrect assignments is significantly lower. This improvement is due to the more careful assignment of short chains. In many cases, introducing stricter rules reduces the numbers of both correct and incorrect assignments of short chains (see, for example, protein 16445 datasets A2 and B2). This is both safe and beneficial: Even if some short chains remain unassigned, reducing the number of errors is advantageous. At the same time, long chains are assigned more efficiently by employing the chemical shift statistics for IDPs (see, for example, protein 19135 dataset B2 and protein 15176 dataset C2). The positive effect of incorporating C^α^ chemical shift statistics for non-glycine residues is reflected in the significant improvement in program’s performance for datasets (A1, B1, C1).

The datasets A for protein 15201 allow us to verify the usefulness of the approach—in particular the IDPs statistics—for urea-unfolded proteins. A total of 87.8 % of residues were correctly assigned for datasets A2 and A3, compared to just 33.3 % for dataset A1. This indicates that the statistical values correctly reflect the chemical shifts of this urea-unfolded protein. In two other urea-unfolded proteins (16626 and 16627), the cross-sections chains were so long that even for dataset A1 almost complete assignment was obtained. For protein 16627, the aliphatic chemical shifts appear to be consistent with the statistics used by TSAR, but for protein 16626 we observed a reduction in the proportion of correct assignments by 1.6 percentage points for datasets containing aliphatic chemical shifts (A2, A3, B2, B3, C2, C3) compared to those lacking such information. The reason for this was the exclusion of the correct amino acid (Asp) from the set of possibilities for one residue. This happened during the first step of amino acid recognition: The C^β^ chemical shift slightly exceeded the range for Asp (BMRB average ± 2 SD). Importantly, the mismatch between the statistics and the real data does not result in incorrect assignment. Overall, therefore, the procedures proposed in this article appear to be applicable for urea-unfolded proteins.

The next question examined during the simulations concerned the amount of information from the 4D γ-selective-HCBCACON experiment versus the 4D HCBCACON. In most cases, the result from datasets A2, B2, and C2 was identical to the results from A3, B3, and C3 respectively. However, in several cases the result was better when using the new γ-selective experiment, inasmuch as the number of correct assignments rose or the number of incorrect assignments fell (e.g. 11526 or 18895 datasets A2–A3). In only one case did replacing the HCBCACON with the γ-selective-HCBCACON decrease the number of correct assignments, namely protein 18580 datasets A2–A3. At the same time, it also removed the incorrect assignment. Overall, therefore, we recommend using the γ-selective-HCBCACON rather than the HCBCACON as it yields extra information without taking extra time.

The simulations indicate that it is usually beneficial to employ amino-acid selective experiments in place of—or in addition to—aliphatic chemical shifts. In twenty-four cases, the result was better for dataset 4 than for the corresponding dataset 3; in only six cases did the opposite apply. In two cases, however, using amino-acid selective experiments as the only source of information on amino acids caused problems. The first case was that of protein 18417 datasets B4 and B5, where an incorrect chain of two cross-sections was mapped. The second case was that of protein 19135 dataset A4, where an incorrect chain constructed of five cross-sections (with a single incorrect link inside it) was mapped. However, in this second case over 68 % of cross-sections were within short chains and the overall result was very low (around 60 % of assigned residues), thus it was a ‘high-risk’ dataset. Overall, therefore, using amino acid-selective experiments appears to be a reliable alternative. Of course, if C^β^ and H^β^ chemical shifts are essential for further studies, the method of choice would be the 4D HCBCACON experiment. But if only the backbone assignment is desired, it is worth considering collecting amino acid-selective 2D spectra.

As shown by datasets 5 and 6, the completeness of the resonance assignment falls when the amount of amino acid-selective data is decreased, although the longer the cross-sections chains, the less visible this effect. The proportion of cross-sections in short chains is therefore a very important parameter for suggesting how many amino acid selective experiments are worth acquiring. Clearly, when making the selections for a particular sample, the protein’s sequence should be considered: It is more valuable to identify the amino acids that are abundant in a given molecule. By comparing datasets 5 and 6, we hoped to identify whether it is more beneficial to acquire more amino-acid selective experiments but only in the CACON version (datasets 5), or fewer selections but in both the CACON and the CANCO versions (datasets 6). Regrettably, no definite conclusion could be drawn here: In seven cases the result was higher for dataset 5, and in eight cases it was higher for dataset 6.

Overall, the results are satisfactory. For 11 proteins, over 95 % of residues whose chemical shifts were deposited in BMRB were correctly assigned. Only in the case of one protein was the proportion below 90 %. Incorrect assignments were rare: They occurred for seven proteins in datasets A (with one linking experiment), four proteins in datasets B (with two linking experiments), and two proteins in datasets C (with three linking experiments). Importantly, the assignment result can usually be increased manually: TSAR provides information that allows the user to get back to doubtful fragments of spectra easily and complete the assignment process. At the same time, incorrect assignments can be identified; a manual inspection is always recommended for short chains. In the simulations, almost all incorrect assignments occurred for short chains or at the very end of long chains, which is relatively easy to spot during manual inspection of the result. The only cases of incorrect assignment for long chains were protein 19135 dataset A4, as mentioned above, and protein 11526 dataset A2 and A5 (4 cross-sections-long chain). In the case of protein 11526, the reason was the very untypical (56.52 ppm) C^α^ chemical shift of 21Val residue.

### Experimental results

The new methods of amino acid recognition were also tested on α-synuclein protein, using ^13^C- or ^1^H-detected basis and high-dimensional experiments. In the case of ^13^C-detected data, amino acid recognition was achieved by using 4D HCBCACON or 4D γ-selective-HCBCACON experiments, or by using the 2D amino-acid selective experiments (eight selections: A, D, E, G, N, Q, S, and FHYW, each in both the (CA)CON and (CA)NCO versions). For ^1^H-detected data, amino acid recognition was achieved only using 2D amino-acid selective experiments; no experiment providing C^β^ and H^β^ chemical shifts was acquired in this case.

The selectivity of the amino acid-selective experiments is reported in Table S1 in the Supplementary Material. In all the spectra, only the peaks of the selected amino acid (or amino acids) are present, with few exceptions: In Q-selective 2D (CA)CON and 2D (CA)NCO experiments there is a leakage of N peaks, but in the 2D (CA)NCO experiment they have the opposite sign to the Q peaks and so are very easy to recognize; in E-selective 2D (CA)CON experiment, D peaks appear, but with opposite sign to the E peaks; in G- and S-selective and 2D (CA)NCO experiments, peaks originating form P residues are present, but in the G-selective one they have the opposite sign to the other peaks. TSAR was thus trained to handle such cases. Regarding the completeness of the information, all the expected peaks were retrieved for all the amino acid-selective spectra.

Twelve datasets were constructed out of the ^13^C-detected data (Table 6). Datasets A1–A4 contain only one links-yielding 5D (H)CACON(CA)CON experiment. Datasets B1–B4 include the 5D (H)CACON(CA)CON and 5D HNCACON experiments. Datasets C1–C4 comprise the 5D (H)CACON(CA)CON, 5D HNCACON, and 5D (HCA)CONCACON experiments. Datasets A1, B1, and C1 do not contain any additional information on amino acids. Datasets A2, B2, and C2 use the 4D HCBCACON experiment. Datasets A3, B3, and C3 use the 4D γ-selective-HCBCACON experiment. Datasets A4, B4, and C4 use all 2D amino acid selective experiments, both in the (CA)CON and (CA)NCO versions.

**Table 6:** Datasets of ^13^C-detected experiments

Basis experiment and sequential link-providing experiment(s)	Percentage of cross-sections in chains		Dataset	Experiment(s) providing information on amino acids	Total experiment time (hours)
	Long (≥4)	Short (1–3)			
3D CACON 5D (H)CACON(CA)CON	68.6	31.4	A1	None	67
			A2	4D HCBCACON	95
			A3	4D γ-selective-HCBCACON	95
			A4	All 2D amino acid selective experiments	79
3D CACON 5D (H)CACON(CA)CON 5D HNCACON	78.6	21.4	B1	none	81
			B2	4D HCBCACON	109
			B3	4D γ-selective-HCBCACON	109
			B4	All 2D amino acid selective experiments	93
3D CACON 5D (H)CACON(CA)CON 5D HNCACON 5D (HCA)CONCACON	85.7	14.3	C1	None	152
			C2	4D HCBCACON	180
			C3	4D γ-selective-HCBCACON	180
			C4	All 2D amino acid selective experiments	164

The results obtained by the TSAR program for different datasets of ^13^C-detected experiments are presented in Table 7. The data analysis shows the effectiveness of the approach. Even using a single experiment yielding the sequential connectivities, 83.5 % correct assignments were achieved when combined with amino acid-selective data. Using two linking experiments made it possible to achieve 86.3 % correct assignments and 2.2 % incorrect ones, using a very limited amount of information on amino acid types (glycine recognition based on the peaks’ signs and the CA chemical shifts). Incorporating additional information (CB chemical shifts or amino-acid selective experiments) improved this result further still. For datasets C1–C4, the result was 89.9 % correct assignments and no incorrect ones.

**Table 7:** Automatic assignment results for ^13^C-detected experiments

Dataset	Percentage of correctly/incorrectly assigned residues			
	…1	…2	…3	…4
A…	65.0/0.0 (47.5/7.2)	79.9/0.7 (81.3/0.7)	83.5/2.9	83.5/0.0
B…	86.3/2.2 (70.5/0.7)	87.1/0.7 (90.6/2.9)	87.1/0.7	89.9/3.6
C…	89.9/0.0 (88.5/2.2)	89.9/0.0 (88.5/0.7)	89.9/0.0	89.9/0.0

Datasets A1, B1, C1, A2, B2, and C2 allowed us to compare the performance of the old and the new versions of the TSAR program (data from the other datasets could not be processed by the old version). As with the simulation, the results demonstrate that the new version of TSAR performs better than the old one: In particular, the number of incorrect assignments was lower in four of the datasets. Only in one dataset (B1) the new program yield more incorrect assignments than the old one (2.2 vs. 0.7 %), but here the number of correct assignments was significantly higher (86.3 vs. 70.5 %).

Using a γ-selective-HCBCACON experiment (datasets A3, B3, C3) instead of the standard HCBCACON experiment (datasets A2, B2, C2) does not cause a significant change in the assignment results. For datasets exploiting one linking experiment only, it allows us to significantly increase the number of correct assignments, but at the same time increases the number of errors (datasets A3 vs. A2).

Using amino acid-selective experiments (datasets A4, B4, C4, D4) seems to be a reliable alternative to measuring C^β^ and H^β^ chemical shifts for amino acid recognition. In the case of shorter cross-sections chains, TSAR performs even better than using β chemical shifts (dataset A4 vs. A2 and A3). Nonetheless, for dataset B4 it introduces some erroneous assignments (in short chains and one at the end of a long chain). It is striking that the total experimental time required for a set of 2D amino acid-selective experiments less than half that required for the HCBCACON (12 hours, compared to 28 hours; see Table 6).

Alternatively, ^1^H-detected experiments providing sequential connectivities can be used for assignment, e.g. 5D BT-HN(COCAN)CONNH and 5D BT-(H)NCO(CAN)CONNH experiments (Piai et al. 2014), which require a 3D BT-HNCO as basis spectrum. Eighteen datasets featuring such experiments were constructed in our study (Table 8). Datasets D1–D6 contain the 5D BT-HN(COCAN)CONNH experiment; datasets E1–E6 include the 5D BT-(H)NCO(CAN)CONNH experiment; and datasets F1–F6 contain both experiments. Different datasets contain different combinations of amino-acid selection experiments. Thus, datasets D1, E1, and F1 include all selections (A, D, E, FHYW, G, N, Q, S) in both the (CA)CON and (CA)NCO versions; datasets D2, E2, and F2 contain the same selections but only in the (CA)CON version; datasets D3, E3, and F3 comprise A, E, G, FHYW, and Q selections in both the (CA)CON and (CA)NCO versions; datasets D4, E4, and F4 include A, E, G, FHYW, and Q selections but only in the (CA)CON version; datasets D5, E5, and F5 include A, E, and G selections in both the (CA)CON and (CA)NCO versions; and datasets D6, E6, and F6 contain A, E, and G selections, but only in the (CA)CON version.

**Table 8:** Datasets of ^1^H-detected experiments

Basis experiment and sequential link-providing experiment(s)	Percentage of cross-sections in chains…		Dataset	Experiment(s) providing information on amino acids	Total experiment time (hours)
	long (≥4)	short (1–3)			
3D BT-HNCO 5D BT-HN(COCAN)CONNH	60.9	39.1	D1	All selections, (CA)CON and (CA)NCO	26
			D2	All selections, only (CA)CON	20
			D3	A, E, G, FHYW, Q selections, (CA)CON and (CA)NCO	22.25
			D4	A, E, G, FHYW, Q selections, only (CA)CON	18
			D5	A, E, G selections, (CA)CON and (CA)NCO	18.5
			D6	A, E, G selections, only (CA)CON	16.25
3D BT-HNCO 5D BT-(H)NCO(CAN)CONNH	82.7	17.3	E1	All selections, (CA)CON and (CA)NCO	26
			E2	all selections, only (CA)CON	20
			E3	A, E, G, FHYW, Q selections, (CA)CON and (CA)NCO	22.25
			E4	A, E, G, FHYW, Q selections, only (CA)CON	18
			E5	A, E, G selections, (CA)CON and (CA)NCO	18.5
			E6	A, E, G selections, only (CA)CON	16.25
3D BT-HNCO 5D BT-HN(COCAN)CONNH 5D BT-(H)NCO(CAN)CONNH	84.2	15.8	F1	All selections, (CA)CON and (CA)NCO	37
			F2	All selections, only (CA)CON	31
			F3	A, E, G, FHYW, Q selections, (CA)CON and (CA)NCO	33.25
			F4	A, E, G, FHYW, Q selections, only (CA)CON	29
			F5	A, E, G selections, (CA)CON and (CA)NCO	29.5
			F6	A, E, G selections, only (CA)CON	27.25

The results provided by the TSAR program using these datasets are summarized in Table 9. Here, again, the robustness of the assignment procedure is confirmed. While using all amino acid selection experiments, the percentage of correct assignments obtained using just a single 5D experiment to establish sequential correlations was 79.9 % for BT-HN(COCAN)CONNH (dataset D1) and 88.8 % for BT-(H)NCO(CAN)CONNH (dataset E1). It is worth mentioning that incorrect assignment (one residue within a short chain) occurred only for datasets A, where the cross-sections chains were shortest.

**Table 9:** Automatic assignment results for ^1^H-detected experiments

Dataset	Percentage of correctly/incorrectly assigned residues					
	…1	…2	…3	…4	…5	…6
D…	79.9/0.7	76.1/0.7	73.9/0.7	55.2/0.7	55.2/0.0	39.6/0.0
E…	88.8/0.0	82.8/0.0	86.6/0.0	80.6/0.0	67.2/0.0	64.2/0.0
F…	91.8/0.0	91.8/0.0	90.3/0.0	90.3/0.0	90.3/0.0	90.3/0.0

## Conclusions

In this study we present a refined version of the automatic resonance assignment TSAR program, with improved assignment efficiency and reliability for IDPs. The changes were made with particular objectives in mind: to exploit peaks’ signs depending on the originating residue; to employ the chemical shift statistics established especially for IDPs; and to analyze the data from amino acid-selective experiments. Besides improving the TSAR program, we also propose a modification of an existing 4D HCBCACON experiment so that information on the amino acid type is coded in the peak sign. These methods were tested in simulations using 16 disordered proteins from the BMRB data base, and then verified experimentally using α-synuclein, a 140-amino acids-long IDP, for both proton- and carbon-detected experiments.

The analysis shows that incorporating the above methods significantly improves the results of the assignment, especially for datasets in which the cross-sections chains are relatively short. Amino acid-selective experiments, which are relatively quick when performed using non-uniform sampling, can be used as an alternative to amino acid recognition based on chemical shift analysis. The proposed methods facilitate the resonance assignment of IDPs and make it both more reliable and more complete.

## Electronic supplementary material

Below is the link to the electronic supplementary material.

Supplementary material 1 (PDF 786 kb)
