# Supplementary material for: Amino acid recognition for automatic resonance assignment of intrinsically disordered proteins
Source: J Biomol NMR. 2016 Feb 18;64:239–53. doi: 10.1007/s10858-016-0024-2 (PMC4824835; doi:10.1007/s10858-016-0024-2)
Supplement: Supplementary file 1 — Supplementary material 1 (PDF 786 kb) [file 10858_2016_24_MOESM1_ESM.pdf]

Amino acid recognition for automatic resonance assignment of intrinsically disordered proteins

Alessandro Piai [1], Leonardo Gonnelli [1], Isabella C. Felli [1], Roberta Pierattelli [1], Krzysztof Kazimierczuk [2], Katarzyna Grudziąż [3], Wiktor Koźmiński [3], Anna Zawadzka-Kazimierczuk [3] ✉

[1] CERM, Department of Chemistry, University of Florence, Via Luigi Sacconi 6, 50019 Sesto Fiorentino (Florence), Italy
[2] Centre of New Technologies, University of Warsaw, Banacha 2C, 02-097 Warsaw, Poland
[3] Biological and Chemical Research Centre, Faculty of Chemistry, University of Warsaw, Żwirki i Wigury 101, 02-089 Warsaw, Poland
✉ e-mail: anzaw@chem.uw.edu.pl

Supplementary Material

NMR experiments

In this section the pulse scheme for the 4D γ-selective-HCBCACON experiment is described.

The arrows indicate the switching of the ^{13}C carrier frequency. Narrow and wide symbols stand for 90° and 180° pulses, respectively. The pulses were applied along the *x* axis unless noted differently. The rectangles represent ^{15}N and ^{1}H non-selective pulses, the round shapes represent band-selective ^{13}C pulses. The grey round shape represents a ^{13}C adiabatic pulse. The two different variants necessary for the IPAP virtual carbonyl decoupling approach are shown. The line denoted with PFG stands for pulsed field gradients applied along the z-axis. All gradients employed had 1 ms of duration and a sine-shape. The phase cycle, the position of the carriers and the duration of the delays for the experiment are reported in the caption of the figure describing the pulse sequence.

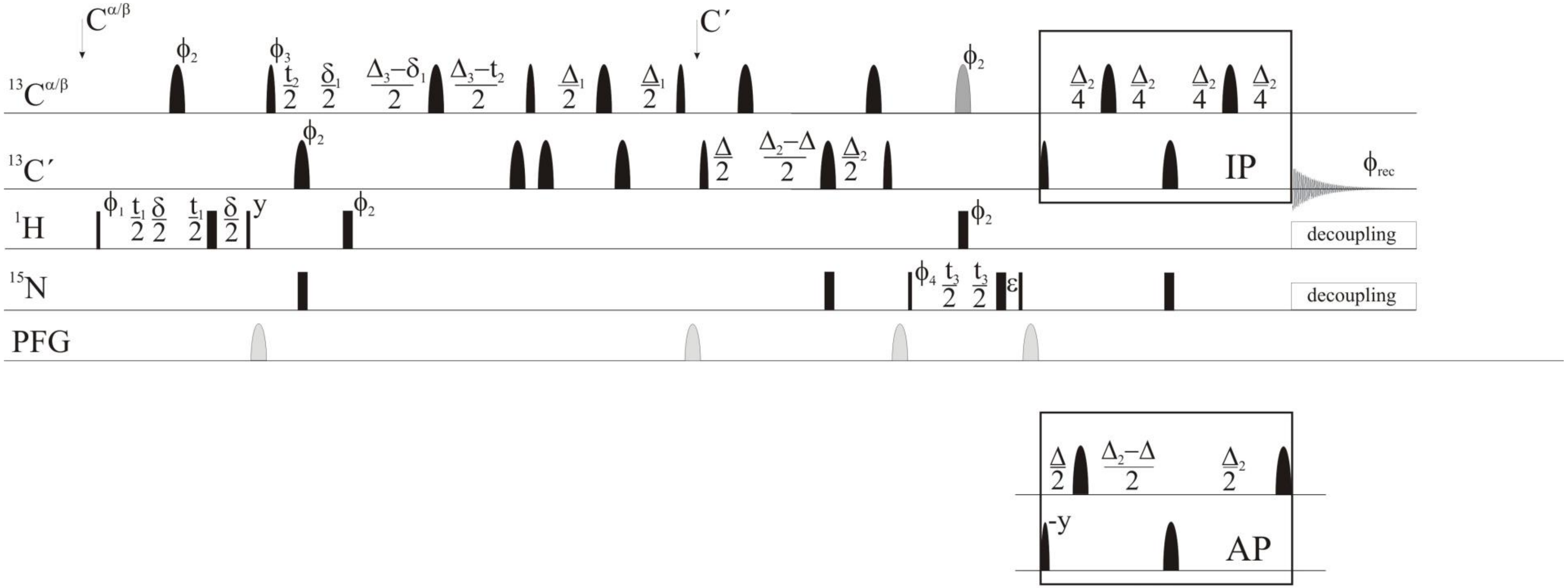

Figure S1. Pulse sequence of the 4D γ-selective-HCBCACON experiment

The following phase cycling was employed: ϕ_1 = x, -x; ϕ_2 = 8(x), 8(-x); ϕ_3 = 2(x), 2(-x); ϕ_4 = 4(x), 4(-x); ϕ_{rec} = x, 2(-x), x, -x, 2(x), -x. The lengths of the delays were: δ = 3.6 ms, δ_1 = 2.2 ms, Δ = 9.0 ms, Δ_1 = 8.0 ms, Δ_2 = 30.0 ms, Δ_3 = 21.0 ms, ε = $t_3(0)+p_8$, where p_8 is the duration of the adiabatic pulse on ^{13}C in the middle of the ^{15}N evolution period. The constant time mode for the evolution of chemical shift was used in aliphatic carbon evolution periods, whereas real time evolution was adopted for proton and nitrogen evolution periods. Quadrature detection in the indirect dimensions was achieved by incrementing phases ϕ_1, ϕ_3 and ϕ_4 in a States manner.

Experimental parameters used. The 4D γ-selective-HCBCACON experiment was recorded in 1 day and 13 hours with 4 scans per increment, an inter scan delay of 1.1 s, spectral widths of 8800 (aq) × 2550 (^{15}N) × 12500 ($^{13}C^{\alpha,\beta}$) × 5000 (^{1}H) Hz with 512 complex points in the acquisition dimension and 1540 hypercomplex points randomly distributed in the indirectly detected dimensions (data points density of 0.051 %). The maximum evolution times in the indirectly detected dimensions were set to 20.5 ms for the $^{13}C^{\alpha,\beta}$ dimension, to 60.4 ms for the ^{15}N dimension and to 15.0 ms for the ^{1}H dimension.

Format of the TSAR program input files

The detailed manual to the TSAR program can be downloaded together with the program from the website http://nmr.cent3.uw.edu.pl/software. TSAR accepts data from almost any experiment. The experiments should be defined in the 'input.txt' file, as shown in Figure S2.

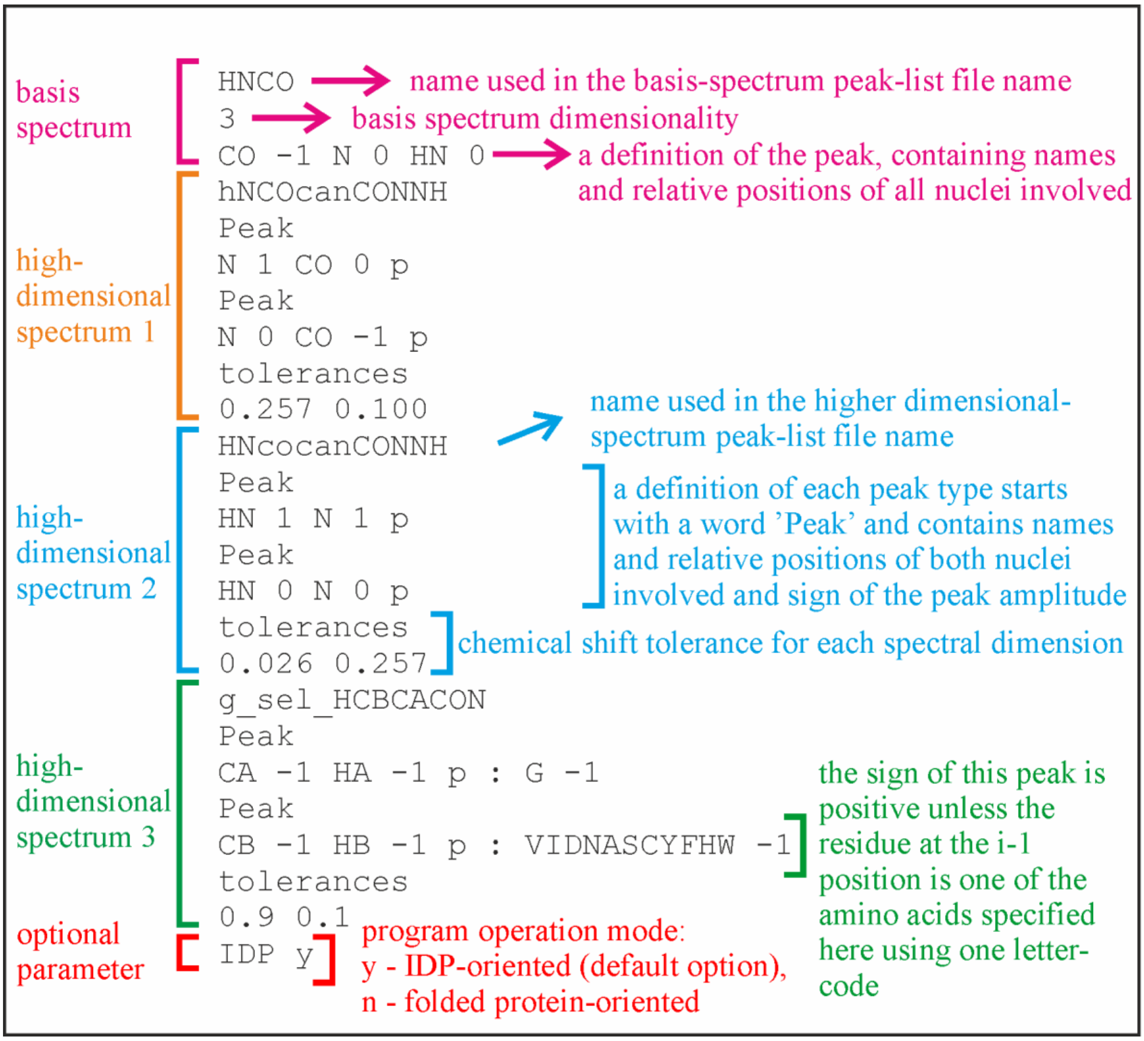

Figure S2. An example of the 'input.txt' file, together with the file format description (color signs). The high-dimensional experiments used in the example are BT-(H)NCO(CAN)CONNH and BT-HN(COCAN)CONNH (*J Biomol NMR* 60 (2014) 209-218) and the new γ-selective-HCBCACON.

In order to use the data from amino acid-selective (CA)CON and (CA)NCO experiments, the 'aa_select.txt' file has to be created. The format of such a file is described below. Incorporating of such an input file for TSAR is optional.

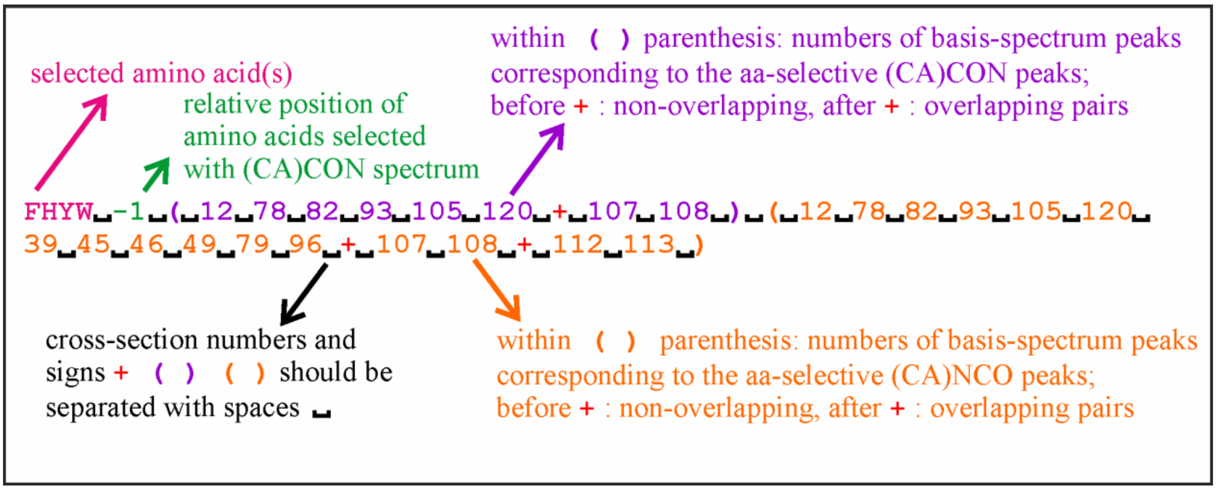

Figure S3. Description of the 'aa_select.txt' file format. The file contains information on the numbers of peaks corresponding to the selected amino acids. In the figure a single line (for one amino acid selection) is shown.

```
N -1 ( 53 61 66 ) ( 53 61 66 32 38 68 )
D -1 ( 32 44 48 49 72 136 ) ( 32 44 48 49 72 136 33 58 60 64
123 140 )
QN -1 ( 11 53 61 66 64 71 85 91 + 80 81 ) ( 53 61 66 64 71
85 91 32 38 68 44 47 59 65 67 82 + 80 81 )
E -1 ( 10 14 60 67 110 116 134 137 + 8 9 + 69 70 ) ( 10 14
60 67 110 116 134 137 40 51 57 61 66 73 76 90 119 120 + 8 9
+ 69 70 + 80 81 + 107 108 )
FHYW -1 ( 12 78 82 93 105 120 + 107 108 ) ( 12 78 82 93 105
120 39 45 46 49 79 96 + 107 108 + 112 113 )
G -1 ( 3 22 24 34 35 36 37 42 43 45 46 52 54 62 101 111 115
) ( 3 22 24 34 35 36 37 42 43 45 46 52 54 62 101 111 115 1 2
4 5 10 11 12 13 14 15 16 17 + 6 7 + 8 9 )
A -1 ( 1 2 18 19 39 40 41 47 50 51 55 57 86 87 92 94 98 135
) ( 1 2 18 19 39 40 41 47 50 51 55 57 86 87 92 94 98 135 101
111 114 115 116 126 127 128 129 130 133 134 + 117 118 + 131
132 )
S -1 ( 83 90 95 97 ) ( 83 90 95 97 22 24 26 27 )
```

Figure S4. Example of the 'aa_select.txt' file. The sample file contains information on eight different amino acid selections, all in both (CA)CON and (CA)NCO versions. Due to not full selectivity of the Q-selective spectrum (see Table S1), the selection was defined as 'QN', instead of 'Q'.

Table S1 The selectivity of 2D amino-acid selective experiments.

selection	(CA)CON	(CA)NCO
	Selectivity	selectivity
A	no u. p. [a]	no u. p.
D	no u. p.	no u. p.
E	D peaks (of opposite sign)	no u. p.
FHYW	no u. p.	no u. p.
G	no u. p.	P peaks (of opposite sign)
N	no u. p.	no u. p.
Q	N peaks	N peaks (of opposite sign)
S	no u. p.	P peaks

[a] no unexpected peaks (peaks of different amino acids)
